# Optical‐controlled magnon transport based on spin crossover switched molecular magnets

**DOI:** 10.1002/smo2.70047

**Published:** 2026-04-27

**Authors:** Yu‐Jing Gao, Shuo Chang, Liang Zhao, Yin‐Shan Meng, Zhiyong Qiu, Tao Liu

**Affiliations:** ^1^ State Key Laboratory of Fine Chemicals, Frontier Science Center for Smart Materials, School of Chemical Engineering Dalian University of Technology Dalian China; ^2^ Key Laboratory of Materials Modification by Laser, Ion, and Electron Beams (Ministry of Education), School of Materials Science and Engineering Dalian University of Technology Dalian China; ^3^ Liaoning Binhai Laboratory Dalian China

**Keywords:** magnon transport, optical‐control, spin crossover, spintronics

## Abstract

Molecule‐based devices that combine the advantages of fast time response and extremely low manipulation/transmission energy consumption of light, as well as the non‐volatile properties of magnetic storage, could potentially be the ideal choice for future information processing. The key to achieving this vision lies in the bridge between light and magnetism, which refers to the innovative magneto‐optical functional materials. The discovery of molecular magnets with spin‐crossover features provides a new inspiration for realizing magneto‐optical fusion information technology. Here, we demonstrate that light can reversibly modulate the propagation of magnons in cyanide bridged alternating W(V)‐Fe(II) coordination polymer chains, wherein the paramagnetic high‐spin and diamagnetic low‐spin states of Fe(II) ions can be interconverted by alternating 808‐ and 473‐nm light irradiations. Our experiments exploit microwaves for spin injection and detection, revealing that characteristic signal peaks at 8.28–8.60 GHz can be modulated by alternating light irradiation. The experimental results relate this phenomenon to the difference in magnon excitation between different spin states resulting from photo‐induced spin‐state switching. This photo‐modulated spin transport device, which exhibits the properties of nonvolatility and reproducibility, provides a revolutionary strategy for modulating magnons and paving the way for optically tunable, ultrafast, low‐power, and organic‐insulator‐based spin‐logic devices.

## Graphical abstract

A cyanide‐bridged W(V)–Fe(II) spin‐crossover single crystal was integrated into a magnon transport device, enabling reversible optical control of magnon transport through photo‐switched spin states.

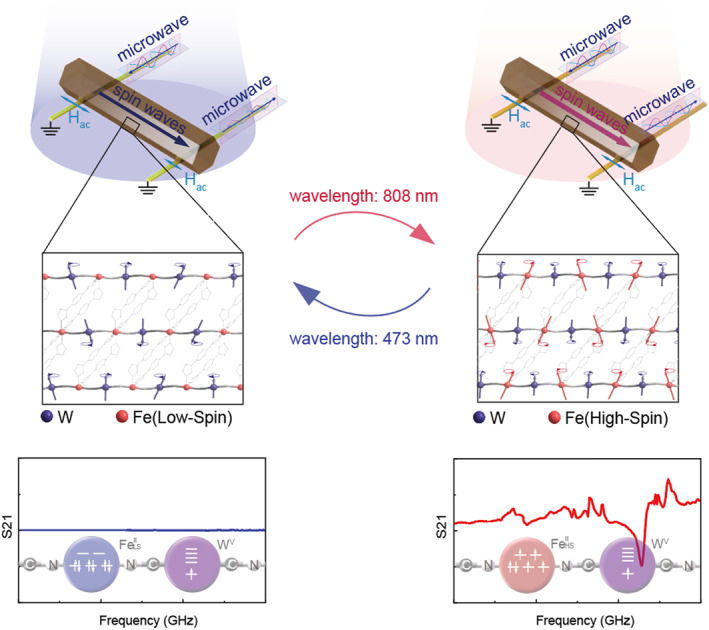

## 1 INTRODUCTION

Photon computers, with their ultra‐fast response times, broad transmission bandwidth, and extremely low energy consumption, are viewed as a potential replacement for electronic computers.[^1^, ^2^, ^3^, ^4^, ^5^, ^6^, ^7^] However, despite decades of research, the concept of the photon computer largely remains theoretical due to numerous technical challenges. One notable issue is the absence of efficient light‐controlled storage devices, which hinders the practical realization of photon computers.^[8]^ On the other hand, storage devices that rely on magnetism and spin are considered to be an ideal solution for information storage due to their non‐volatility, speed, and ease of integration.[^9^, ^10^, ^11^, ^12^, ^13^] Despite this, their commercial development is seemingly stymied by complex manufacturing processes and high operational energy consumption. It stands to reason, therefore, that the combination of the speed and energy advantages of photon‐based information transmission, with the non‐volatility and quick response of spin‐based systems, presents a valuable and prospective research direction for information storage.[^14^, ^15^, ^16^] The primary challenge lies in developing light‐magnetic functional materials and designing devices based on light‐magnetic fusion concepts.

**FIGURE 1:**
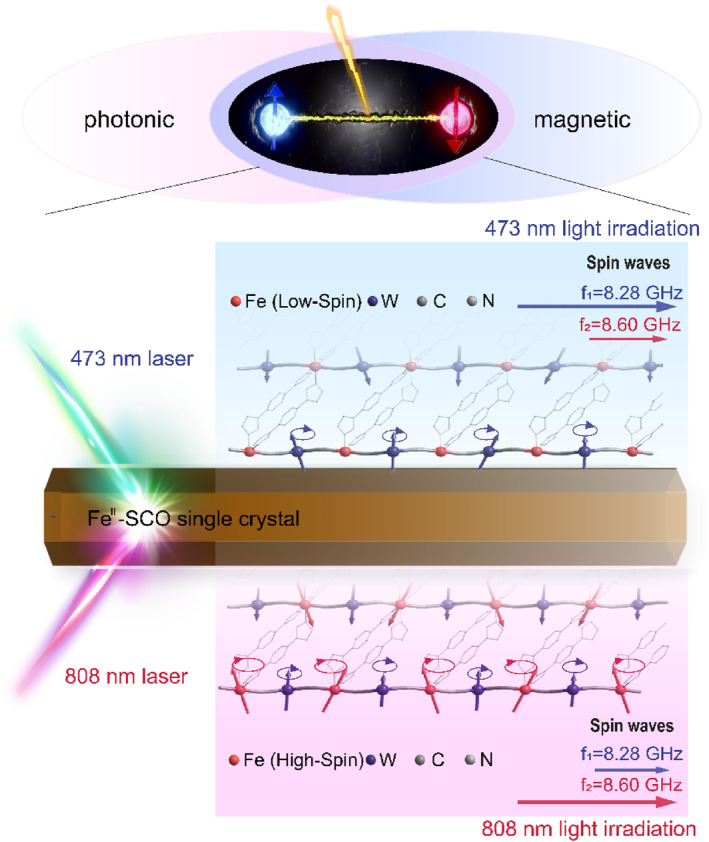
Schematic illustration of the principle of optical actuation different magnon propagation based on different spin states in Fe^II^‐SCO. Fe^II^‐SCO, a magnetic insulator with opto‐magnetic effect, can reversibly control the spin state by a laser to control the spin waves’ transmission. Spin waves with characteristic frequency *f*_1_ = 8.28 GHz are generated by microwave excitation in a W^V^‐Fe^II^ single crystal under 473 nm light irradiation, and those with characteristic frequency *f*_2_ = 8.60 GHz under 808 nm light irradiation.

Commonly, light‐magnetism coupling effects, such as the magneto‐optical Kerr effect[^17^, ^18^, ^19^, ^20^] or Brillouin scattering,[^21^, ^22^, ^23^, ^24^, ^25^, ^26^, ^27^] could potentially be utilized for correlating and converting a material's photonic/magnetic properties. However, the application of these foundational physical effects, and their inverse effects, often present issues such as weak magnetic coupling, low conversion efficiencies, and large energy consumption.[^28^, ^29^, ^30^, ^31^] This makes the design and implementation of practical photo‐magnetic storage devices particularly challenging. The recent discovery and research of switchable molecular magnets, particularly crystals with photo‐responsive spin‐crossover (SCO) properties,[^32^, ^33^, ^34^, ^35^, ^36^, ^37^, ^38^, ^39^, ^40^] have provided new perspectives for the development of photon‐magnetic coupling devices. Spin‐crossover materials can facilitate high‐efficiency, low‐energy, and high‐speed conversions between light and magnetism to enable high‐density information storage at a molecular scale (Figure 1). Additionally, the variety of spatial dimensional configurations related to spin transport properties may open up numerous possibilities for the construction of logic devices with complex information interaction channels.

## 2 RESULTS AND DISCUSSION

**FIGURE 2:**
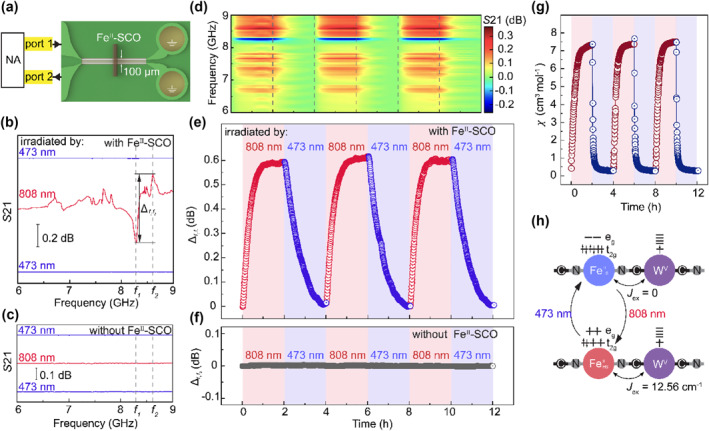
Light modulation of magnon propagation in Fe^II^‐SCO crystal. (a) Schematic illustration of the device structure. The microwave spectrum in the 6–9 GHz range after 808‐(red line) and 473‐nm (blue line) light irradiation at 10 K of the crystal‐device (b) and crystal‐free‐device (c). The test is for the curve when the laser is turned off. The curves with the 808 nm laser on and the 808 nm laser off are depicted in Supporting Information S1: Figures S3 and S4. (d) The time–frequency characteristics of the signal under alternating 808‐ and 473‐nm‐light modulations. The intensity of the peaks is depicted through a rainbow color scale, where positive values are colored in warm hues, and negative values are colored in cool hues. The time‐dependent Δ_*f*1*f*2_ by alternating 808‐ and 473‐nm light irradiation at 10 K of the crystal‐device (e) and crystal‐free‐device (f). (g) The time‐dependent magnetic susceptibility by applying 808‐ and 473‐nm light irradiation at 10 K. (h) Schematic illustration of the reversible photo‐switched spin state of Fe^II^ centers and photo‐switchable magnetic interactions between Fe^II^ and W^V^ ions.

This study investigated the influence of light irradiation on magnon propagation in Fe^II^‐SCO single crystal using the experimental setup shown in Figure 2a and Figure S2. The single crystal was set on two parallel antennas, and the molecular chain was perpendicular to the two antennas in the direction of spin‐wave propagation. Lasers of 808 and 473 nm wavelengths were utilized to switch the iron ions between Fe^II^_HS_ and Fe^II^_LS_ in Fe^II^‐SCO crystal.

The magnon in Fe^II^‐SCO crystal was excited by microwave. The magnetic field component of microwaves (*H*_microwave_) can interact with the magnetic moments (*M*) in magnetic materials. When the frequency of the microwave (*f*_microwave_) matches the resonance frequency of the magnetic moments (*f*_res_), the magnetic moments resonate and precess, which can be described by the Landau–Lifshitz–Gilbert equation. This precession can propagate through the magnetic material via magnetic dipole interactions, giving rise to magnons.

Concurrently, S21 scattering parameters were measured in the 6–9 GHz frequency band through a network analyzer (NA). The excitation and propagation of magnons alter the propagation path and phase of the microwave in the material, leading to changes in the S21 parameter. The S21 parameter, in this context, represents the transmission properties of microwave‐excited magnons within Fe^II^‐SCO crystal. The sample was initialized at the onset using 473 nm blue light, where the Fe^II^ ions were in the low‐spin (LS) state. All S21 data were normalized by considering the signal taken after initializing as the reference (Figure 2b). Upon irradiation by 808 nm red light, several characteristic signal peaks appeared, which disappeared after irradiation with 473 nm blue light again (Figure 2b). Two significant signal peaks appeared at *f*_1_ = 8.28 GHz and *f*_2_ = 8.60 GHz, which may correspond to the most effective frequency for magnon transmission after irradiation by different lights. It is worth noting that the characteristic signals remain even after turning off the 808 nm light source, indicating that the signals are non‐volatile and ruling out the photo‐thermal effect (Supporting Information S1: Figures S3 and S4). No change in the S21 parameters was observed for the blank measurement under the same irradiation condition without Fe^II^‐SCO crystal (Figure 2c). These results strongly indicate that the variation in microwave spectra shown in Figure 2b is due to the response of the Fe^II^‐SCO crystal to light excitation. Specifically, the excitation of light modulates the transport of magnons in the crystal, and this phenomenon is reversible and non‐volatile.

The time‐frequency characteristic of the signal under light modulation exhibits excellent repeatability (Figure 2d). The frequency and intensity of the signal peaks agree well with those in Figure 2b. During the first light irradiation by 808 nm for 2 h, peaks appeared at 8.28 and 8.60 GHz, corresponding to the extrema of 0.35 and −0.25 dB, respectively. Upon altering irradiation with 473 nm light, those peaks gradually decreased and disappeared entirely in about 2 h. Excitingly, the signal peaks were completely reproduced at the same position and with the same intensity by the second 808 nm light irradiation, and it confirmed the reproducible signal amplitude upon the alternative irradiations of 808 and 473 nm lights. This suggests that optical modulation of magnon propagation has a broad frequency response.

Figure 2e represents the time‐dependent behavior of Δ_*f*1*f*2_ by altering the irradiation light between 808 and 473 nm on the Fe^II^‐SCO crystal, where Δ_*f*1*f*2_, referring to the difference between the microwave signal S21 intensity at 8.60 and 8.28 GHz (Figure 2b), serves as a figure of merit for quantifying the switching effect. Upon the 808 nm laser irradiation, the Δ_*f*1*f*2_ value increases gradually, reaching a saturated value of about 0.6 dB after 2 h. Conversely, under irradiation with a 473 nm laser, Δ_*f*1*f*2_ slowly decreased and disappeared entirely after approximately 2 h. Despite a maximum value fluctuation of 0.05 dB due to background noise, the Δ_*f*1*f*2_ value underwent predictable and repeatable changes upon the alternating light irradiation. At the same time, the blank experiment without the Fe^II^‐SCO crystal showed no changes in the Δ_*f*1*f*2_ under the same irradiation condition (Figure 2f). It is reiterated that the Fe^II^‐SCO crystal is solely responsible for all microwave signal variations and that the photo‐switching behavior highlights the high stability and repeatability of the signal. Note that due to the current bulk material morphology, the HS ↔ LS transition does not occur instantaneously in comparison with those at the molecular or single‐layer scale. As a result, the device's real‐world functionality appears as a continuous modulation of signals at the two characteristic frequencies, 8.60 and 8.28 GHz, respectively. As such, the device can be conceptualized as a binary device.

Magnons’ transport relies on the magnetic exchange interactions within the material, which enable magnons to propagate over long distances in a material. The Fe^II^‐SCO complex shows reversible light‐induced spin state conversions between paramagnetic Fe^II^_HS_ and diamagnetic Fe^II^_LS_ states, called light induced excited spin state trapping (LIESST)[^37^, ^41^]; therefore, the variation in Δ_*f*1*f*2_ is corresponsible for the change in the intrinsic magnetic properties of the material upon the light irradiation. Consistent variations were also observed in Δ_*f*1*f*2_ and the magnetic susceptibility data when 808 and 472 nm lights irradiated the samples in the same sequence (Figure 2g). The longer irradiation time is due to the transition probability and strong absorbance characteristics of the bulk crystal^[42]^. As a consequence, this transition is not achieved in the picosecond regime, a finding consistent with related SCO photomagnetic research.[^32^, ^34^, ^35^, ^43^, ^44^] Nonetheless, the consistency of photomagnetic and photo‐modulated magnon transport experiments indicates that the propagation characteristics of magnons are associated with the magnetic properties of the Fe^II^‐SCO crystal upon light irradiation (Figure 2h). In the LS state of Fe^II^‐SCO, the W^V^ (*S* = 1/2) ions are magnetically separated by the diamagnetic Fe^II^ ions. In contrast, in the high‐spin (HS) state, the paramagnetic Fe^II^_HS_ (*S* = 2) and W^V^ ions are alternately aligned in the chain with the estimated magnetic exchange interaction of *J* = +12.56 (3) cm^−1^ (Figure 2h), leading to the mode changing of the propagation magnon in the Fe^II^‐SCO crystal.

**FIGURE 3:**
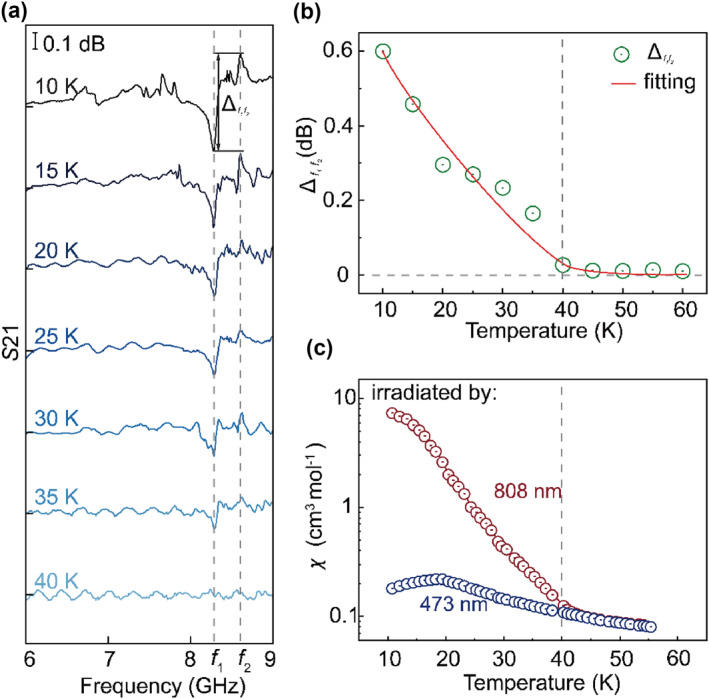
Temperature‐dependent photoinduced magnon modulation. (a) The microwave spectrum at various temperatures after 808‐nm‐light irradiation. (b) The temperature‐dependent Δ_*f*1*f*2_. (c) The temperature‐dependent susceptibilities after 808‐ and 473‐nm‐light irradiation.

The microwave spectra measured at different temperatures further certify that the magnon propagation modulated by light originated from variations of magnetic properties in Fe^II^‐SCO (Figure 3). Similar peaks at frequencies of *f*_1_ and *f*_2_ were observed in microwave spectra at different temperatures. The Δ_*f*1*f*2_ values decreased gradually from the maximum value of about 0.6 dB at 10 K, eventually merging into noise at around 40 K (Figure 3b). The temperature variation of Δ_*f*1*f*2_ value is consistent with those of the magnetic susceptibility data (Figure 3c). The characteristic temperature of 40 K, magnetic data of HS and LS states merging into one, corresponds to the critical temperature at which the photo‐generated HS state thermally relaxes to the LS state. As such, photo‐modulated magnon transport experiment was conducted at 10 K to ensure the stability and longevity of the photoinduced HS state of Fe^II^ in the LIESST regime. It is worth noting that several other Fe^II^‐SCO complexes showing the relaxation temperature of LIESST at liquid nitrogen temperature.[^45^, ^46^, ^47^, ^48^] And metal‐to‐metal electron transfer systems, such as certain FeCo Prussian blue analogs with *T*_LIESST_ up to 200 K,^[49]^ showcase promising photo‐switching properties for room‐temperature operation. These remarkable photo‐switching properties highlight their potential in room‐temperature operation of magnon devices.

## 3 CONCLUSION

In summary, we developed a nonvolatile and reversible light‐controlled spin transport device using Fe^II^‐SCO crystals. By alternating the excitation light, the spin state of the spin state of Fe^II^‐SCO crystal is converted between HS and LS states, which in turn affects the magnon excitation within it. As a result, modulation of spin transport at characteristic microwave frequencies is accomplished. Our experiments validated that this phenomenon is associated with the photo‐excited SCO effect in Fe^II^‐SCO crystals. We propose a prototype of this optical‐controlled organic magnon transport, thus facilitating the potential diversification of spin manipulation. The prospect of optical modulation of magnon may radically transform data storage and information processing technologies, owing to significant benefits such as optical tunability, ultrafast and nonvolatile nature, low power consumption, and high data processing speeds.

## 4 EXPERIMENTAL SECTION/METHODS

### 4.1 Synthesis of the Fe^II^‐SCO single crystal

The single crystal of compound [W^V^(CN)_8_][(Fe^II^)(bib)_2_](bibH) 2CH_3_OH was synthesized with the liquid phase diffusion method according to the modified procedures in the literature.^[36]^ An aqueous solution that contained Fe(ClO_4_)_2_·6H_2_O was placed at the bottom of a test tube, a buffer solution mixture of methanol and water (1:1, v/v) was gently layered on the top of the solution, and then, methanol solution that contained (Bu_4_N)_3_[W(CN)_8_]·2H_2_O and the 1,4‐bis(1H‐imidazol‐1‐yl) benzene ligand was added as the third layer. The crystals were obtained on the tube wall in the middle of the test tube after several weeks. Crystal structure of Fe^II^‐SCO is depicted in Supporting Information S1: Figure S1.

### 4.2 Details of the microwave‐transport measurement

The magnon transport measurement was carried out in a cryogenic environment. A NA was used as a microwave source and a detector, which was set to work in NA mode. The frequency range and the scattering parameters (S‐parameters) were set as 6–9 GHz and S21, respectively. The crystal was set on a coplanar waveguide with a pair of microstrip antennas; the two antennas were separately connected to Port 1 1 and Port 2 of the NA, and their grounds were connected at the other ends so that the electric field component was nearly zero at the ends of the two antennas. Millimeter‐scale samples and a waveguide configuration were utilized. Specifically, the Fe(II)‐SCO single crystal is shaped as a hexagonal prism with a diameter of approximately 0.2 mm and a length of about 2 mm. The microwave antennas used for excitation and detection are spaced 0.1 mm apart (Supporting Information S1: Figure S2). A maximum alternative magnetic field was then excited near the crystal, which allowed us to maximize the intensity of excited magnons. The crystal was irradiated by lasers with two wavelengths (808 nm, 473 nm) selectively during the measurement. The laser lights were separately derived by two programmable laser devices and were transmitted to the crystal through an optical fiber. For optical power density measurements, light was introduced via optical fiber into the low‐temperature chamber and directed onto the Fe(II)‐SCO crystal through a collimating lens, resulting in a power density of approximately 0.1 mW/mm^2^. Considering the device's core area of 0.2 × 0.2 mm^2^, the effective optical power required for switching is merely 2 nW.

### 4.3 Photomagnetic measurements

The magnetic properties of the samples were assessed using a SQUID magnetometer. The time‐dependent magnetic susceptibility was measured under a 1000 Oe field at 10 K by alternating a blue (*λ* = 473 nm and 20 mW/cm^2^) and a red (*λ* = 808 nm and 20 mW/cm^2^) lasers. The temperature‐dependent magnetic susceptibility was measured under a 1000 Oe field before and after irradiation in the temperature range from 10 to 55 K.

## CONFLICT OF INTEREST STATEMENT

The authors declare no conflicts of interest.

## ETHICS STATEMENT

This study did not involve human participants, animals, or any data derived from human subjects.

## Supporting information

Supporting Information S1

## Data Availability

The data supporting this article have been included as part of the Supporting Information and can be obtained freely by asking the authors.

## References

[1] B. Hacker , S. Welte , G. Rempe , S. Ritter , Nature 2016, 536, 193. 27383791 10.1038/nature18592

[2] A. Nicolas , L. Veissier , L. Giner , E. Giacobino , D. Maxein , J. Laurat , Nat. Photon 2014, 8, 234. 10.1364/OL.38.000712 23455274

[3] T. Zhong , J. M. Kindem , J. G. Bartholomew , J. Rochman , I. Craiciu , E. Miyazono , M. Bettinelli , E. Cavalli , V. Verma , S. W. Nam , F. Marsili , M. D. Shaw , A. D. Beyer , A. Faraon , Science 2017, 357, 1392. 28860208 10.1126/science.aan5959

[4] J. M. Arrazola , V. Bergholm , K. Brádler , T. R. Bromley , M. J. Collins , I. Dhand , A. Fumagalli , T. Gerrits , A. Goussev , L. G. Helt , J. Hundal , T. Isacsson , R. B. Israel , J. Izaac , S. Jahangiri , R. Janik , N. Killoran , S. P. Kumar , J. Lavoie , A. E. Lita , D. H. Mahler , M. Menotti , B. Morrison , S. W. Nam , L. Neuhaus , H. Y. Qi , N. Quesada , A. Repingon , K. K. Sabapathy , M. Schuld , D. Su , J. Swinarton , A. Száva , K. Tan , P. Tan , V. D. Vaidya , Z. Vernon , Z. Zabaneh , Y. Zhang , Nature 2021, 591, 54. 33658692 10.1038/s41586-021-03202-1 PMC11008968

[5] H.‐S. Zhong , H. Wang , Y.‐H. Deng , M.‐C. Chen , L.‐C. Peng , Y.‐H. Luo , J. Qin , D. Wu , X. Ding , Y. Hu , P. Hu , X.‐Y. Yang , W.‐J. Zhang , H. Li , Y. Li , X. Jiang , L. Gan , G. Yang , L. You , Z. Wang , L. Li , N.‐L. Liu , C.‐Y. Lu , J.‐W. Pan , Science 2020, 370, 1460. 33273064 10.1126/science.abe8770

[6] L. S. Madsen , F. Laudenbach , M. F. Askarani , F. Rortais , T. Vincent , J. F. F. Bulmer , F. M. Miatto , L. Neuhaus , L. G. Helt , M. J. Collins , A. E. Lita , T. Gerrits , S. W. Nam , V. D. Vaidya , M. Menotti , I. Dhand , Z. Vernon , N. Quesada , J. Lavoie , Nature 2022, 606, 75. 35650354 10.1038/s41586-022-04725-x PMC9159949

[7] C. Sun , M. T. Wade , Y. Lee , J. S. Orcutt , L. Alloatti , M. S. Georgas , A. S. Waterman , J. M. Shainline , R. R. Avizienis , S. Lin , B. R. Moss , R. Kumar , F. Pavanello , A. H. Atabaki , H. M. Cook , A. J. Ou , J. C. Leu , Y.‐H. Chen , K. Asanović , R. J. Ram , M. A. Popović , V. M. Stojanović , Nature 2015, 528, 534. 26701054 10.1038/nature16454

[8] K. Heshami , D. G. England , P. C. Humphreys , P. J. Bustard , V. M. Acosta , J. Nunn , B. J. Sussman , J. Mod. Opt. 2016, 63, 2005. 27695198 10.1080/09500340.2016.1148212 PMC5020357

[9] S. Nakatsuji , N. Kiyohara , T. Higo , Nature 2015, 527, 212. 26524519 10.1038/nature15723

[10] A. Hoffmann , S. D. Bader , Phys. Rev. Appl. 2015, 4, 047001.

[11] A. Hirohata , K. Yamada , Y. Nakatani , I.‐L. Prejbeanu , B. Diény , P. Pirro , B. Hillebrands , J. Magn. Magn Mater. 2020, 509, 166711.

[12] S. A. Wolf , D. D. Awschalom , R. A. Buhrman , J. M. Daughton , S. Von Molnár , M. L. Roukes , A. Y. Chtchelkanova , D. M. Treger , Science 2001, 294, 1488. 11711666 10.1126/science.1065389

[13] P. Qin , H. Yan , X. Wang , H. Chen , Z. Meng , J. Dong , M. Zhu , J. Cai , Z. Feng , X. Zhou , L. Liu , T. Zhang , Z. Zeng , J. Zhang , C. Jiang , Z. Liu , Nature 2023, 613, 485. 36653565 10.1038/s41586-022-05461-y

[14] J. Igarashi , W. Zhang , Q. Remy , E. Díaz , J.‐X. Lin , J. Hohlfeld , M. Hehn , S. Mangin , J. Gorchon , G. Malinowski , Nat. Mater. 2023, 22, 725. 36894773 10.1038/s41563-023-01499-z

[15] S. D. Sarma , Rev. Mod. Phys. 2004, 76, 324.

[16] C. Wang , Y. Liu , Nano Converg. 2020, 7, 35. 33170368 10.1186/s40580-020-00246-3 PMC7655883

[17] K. Sato , T. Ishibashi , Front. Phys. 2022, 10, 946515.

[18] T. Higo , H. Man , D. B. Gopman , L. Wu , T. Koretsune , O. M. J. Van’t Erve , Y. P. Kabanov , D. Rees , Y. Li , M.‐T. Suzuki , S. Patankar , M. Ikhlas , C. L. Chien , R. Arita , R. D. Shull , J. Orenstein , S. Nakatsuji , Nat. Photon 2018, 12, 73. 10.1038/s41566-017-0086-z PMC5997294 29910828

[19] J. Qin , S. Xia , W. Yang , H. Wang , W. Yan , Y. Yang , Z. Wei , W. Liu , Y. Luo , L. Deng , L. Bi , Nanophotonics 2022, 11, 2639. 39635688 10.1515/nanoph-2021-0719 PMC11501839

[20] J. Wang , C. Sun , Y. Hashimoto , J. Kono , G. A. Khodaparast , Ł. Cywiński , L. J. Sham , G. D. Sanders , C. J. Stanton , H. Munekata , J. Phys. Condens. Matter 2006, 18, R501.

[21] S. Kojima , Materials 2022, 15, 3518.35629540

[22] F. Kargar , A. A. Balandin , Nat. Photon. 2021, 15, 720.

[23] P. Pirro , V. I. Vasyuchka , A. A. Serga , B. Hillebrands , Nat. Rev. Mater. 2021, 6, 1114.

[24] B. J. Eggleton , C. G. Poulton , P. T. Rakich , Michael J. Steel , G. Bahl , Nat. Photon. 2019, 13, 664.

[25] S. Tacchi , G. Gubbiotti , M. Madami , G. Carlotti , J. Phys. Condens. Matter 2017, 29, 073001. 28008880 10.1088/1361-648X/29/7/073001

[26] A. B. Singaraju , D. Bahl , L. L. Stevens , AAPS PharmSciTech 2019, 20, 109. 30746575 10.1208/s12249-019-1311-5

[27] A. V. Chumak , V. I. Vasyuchka , A. A. Serga , B. Hillebrands , Nat. Phys. 2015, 11, 453.

[28] M. Merklein , I. V. Kabakova , A. Zarifi , B. J. Eggleton , Appl. Phys. Rev. 2022, 9, 041306.

[29] A. Kirilyuk , A. V. Kimel , T. Rasing , Rep. Prog. Phys. 2013, 76, 026501. 23377279 10.1088/0034-4885/76/2/026501

[30] A. Kimel , A. Zvezdin , S. Sharma , S. Shallcross , N. De Sousa , A. García‐Martín , G. Salvan , J. Hamrle , O. Stejskal , J. McCord , S. Tacchi , G. Carlotti , P. Gambardella , G. Salis , M. Münzenberg , M. Schultze , V. Temnov , I. V. Bychkov , L. N. Kotov , N. Maccaferri , D. Ignatyeva , V. Belotelov , C. Donnelly , A. H. Rodriguez , I. Matsuda , T. Ruchon , M. Fanciulli , M. Sacchi , C. R. Du , H. Wang , N. P. Armitage , M. Schubert , V. Darakchieva , B. Liu , Z. Huang , B. Ding , A. Berger , P. Vavassori , J. Phys. D Appl. Phys. 2022, 55, 463003.

[31] A. Kirilyuk , A. V. Kimel , T. Rasing , Rev. Mod. Phys. 2010, 82, 2731.

[32] N. Yao , L. Zhao , H. Sun , C. Yi , Y. Guan , Y. Li , H. Oshio , Y. Meng , T. Liu , Angew. Chem. Int. Ed. 2022, 61, e202208208. 10.1002/anie.202208208 36103279

[33] L. Kipgen , M. Bernien , F. Tuczek , W. Kuch , Adv. Mater. 2021, 33, 2008141. 33963619 10.1002/adma.202008141 PMC11468565

[34] J.‐L. Wang , Q. Liu , Y.‐S. Meng , H. Zheng , H.‐L. Zhu , Q. Shi , T. Liu , Inorg. Chem. 2017, 56, 10674. 28812903 10.1021/acs.inorgchem.7b01633

[35] X. Feng , C. Mathonière , I.‐R. Jeon , M. Rouzières , A. Ozarowski , M. L. Aubrey , M. I. Gonzalez , R. Clérac , J. R. Long , J. Am. Chem. Soc. 2013, 135, 15880. 24066720 10.1021/ja407332y

[36] L. Zhao , Y.‐S. Meng , Q. Liu , O. Sato , Q. Shi , H. Oshio , T. Liu , Nat. Chem. 2021, 13, 698. 34031565 10.1038/s41557-021-00695-1

[37] S. Decurtins , P. Gütlich , C. Köhler , H. Spiering , A. Hauser , Chem. Phys. Lett. 1984, 105, 1.

[38] C. Lefter , S. Rat , J. S. Costa , M. D. Manrique‐Juárez , C. M. Quintero , L. Salmon , I. Séguy , T. Leichle , L. Nicu , P. Demont , A. Rotaru , G. Molnár , A. Bousseksou , Adv. Mater. 2016, 28, 7508. 27308873 10.1002/adma.201601420

[39] X. Xiao , Z. J. Chen , R. J. Varley , C. H. Li , Smart Mol. 2024, 2, e20230028. 40625804 10.1002/smo.20230028 PMC12118250

[40] I. C. Y. Hou , L. Li , H. Zhang , P. Naumov , Smart Mol. 2024, 2, e20230031. 40625528 10.1002/smo.20230031 PMC12118287

[41] A. Hauser , Chem. Phys. Lett. 1986, 124, 543.

[42] A. Marino , M. Servol , R. Bertoni , M. Lorenc , C. Mauriac , J.‐F. Létard , E. Collet , Polyhedron 2013, 66, 123.

[43] S. Ghosh , S. Kamilya , T. Pramanik , M. Rouzières , R. Herchel , S. Mehta , A. Mondal , Inorg. Chem. 2020, 59, 13009. 32875794 10.1021/acs.inorgchem.0c02136

[44] K. Ridier , A. Hoblos , S. Calvez , M. Lorenc , W. Nicolazzi , S. Cobo , L. Salmon , L. Routaboul , G. Molnár , A. Bousseksou , Coord. Chem. Rev. 2025, 535, 216628.

[45] A. Djemel , O. Stefanczyk , C. Desplanches , K. Kumar , R. Delimi , F. Benaceur , S. Ohkoshi , G. Chastanet , Inorg. Chem. Front. 2021, 8, 3210.

[46] V. García‐López , M. Palacios‐Corella , S. Cardona‐Serra , M. Clemente‐León , E. Coronado , Chem. Commun. 2019, 55, 12227. 10.1039/c9cc05988a 31552999

[47] G. Agustí , S. Cobo , A. B. Gaspar , G. Molnár , N. O. Moussa , P. Á. Szilágyi , V. Pálfi , C. Vieu , M. Carmen Muñoz , J. A. Real , A. Bousseksou , Chem. Mater. 2008, 20, 6721.

[48] J. Létard , P. Guionneau , O. Nguyen , J. S. Costa , S. Marcén , G. Chastanet , M. Marchivie , L. Goux‐Capes , Chem. A Eur. J 2005, 11, 4582. 10.1002/chem.200500112 15861388

[49] D. Li , R. Clérac , O. Roubeau , E. Harté , C. Mathonière , R. Le Bris , S. M. Holmes , J. Am. Chem. Soc. 2008, 130, 252. 18076169 10.1021/ja0757632

