# Supplementary material for: Optical‐controlled magnon transport based on spin crossover switched molecular magnets
Source: Smart Mol. 2026 Apr 27;4(2):e70047. doi: 10.1002/smo2.70047 (PMC13317715; doi:10.1002/smo2.70047)
Supplement: Supplementary file 1 — Supporting Information S1 [file SMO2-4-e70047-s001.docx]

Supporting Information

Optical-Controlled Magnon Transport based on Spin Crossover-Switched Molecular Magnet

Yu-Jing Gao^1^, Shuo Chang^2^, Liang Zhao^1^, Yin-Shan Meng^1,3^*, Zhiyong Qiu^2^* and Tao Liu^1,3^*

^1^State Key Laboratory of Fine Chemicals, Frontier Science Center for Smart Materials, School of Chemical Engineering, Dalian University of Technology, Dalian 116024, China.

^2^Key Laboratory of Materials Modification by Laser, Ion, and Electron Beams (Ministry of Education), School of Materials Science and Engineering, Dalian University of Technology, Dalian 116024, China.

^3^Liaoning Binhai Laboratory, Dalian 116023, China.

E-mail: Tao Liu: [liutao@dlut.edu.cn](mailto:liutao@dlut.edu.cn); Zhiyong Qiu: qiuzy@dlut.edu.cn; Yin-Shan Meng: mengys@dlut.edu.cn

[Metric Δ*_f_*_1_*_f_*_2_ 3](#_Toc221314492)

[Calculations information of ferromagnetic coupling 3](#_Toc221314493)

[Figure S1. Crystal structure of Fe^II^-SCO. 4](#_Toc221314494)

[Figure S2. Optical image (a) and schematic illustration (b) of the device structure. 5](#_Toc221314495)

[Figure S3. The microwave spectrum S21 of the crystal-device with the 808 nm laser on and the 808 nm laser off at 10 K. 6](#_Toc221314496)

[Figure S4. The time-dependent Δ*_f_*_1_*_f_*_2_ with the 808 nm laser on and the 808 nm laser off at 10 K of the crystal-device. 7](#_Toc221314497)

# Metric Δ*_f_*_1_*_f_*_2_

In our study, the core mechanism involves the light-induced transition between high-spin (HS) and low-spin (LS) states in Fe(II), which in turn modulates magnon propagation. Intrinsically, this transition creates distinct spin-resonant excited states with different characteristic frequencies. Thus, from an operational standpoint, the device can be conceptualized as a binary ON/OFF switch. Nonetheless, due to the current material morphology and the conditions under which optical excitation is applied, the HS↔LS transition does not occur instantaneously or uniformly. As a result, the device’s real-world functionality appears as a continuous modulation of signals at the two characteristic frequencies, 8.60 GHz and 8.28 GHz. Metric ∆*_f_*_1_*_f_*_2_ represents the S21 intensity difference between these frequencies; it serves as a figure of merit for quantifying the switching effect.

# Calculations information of ferromagnetic coupling

Calculations were performed with SOC included, while allowing full freedom for magnetic moment orientation throughout the self-consistent process. Under these conditions, the ground state was determined automatically. For the nature of the magnetic interactions in the Fe-W chains, we performed the simulation of the magnetic susceptibility with the Hamiltonian ***H*** = $\sum-2J(S_{W_{i}}S_{\mathrm{Fe}_{i}}+S_{W_{i}}S_{\mathrm{Fe}_{i+1}})$. The *χT* vs. *T* plots were fitted using the expression in literatures (Magnetism: Molecules to Materials I: Models and Experiments, ed. J. S. Miller, M. Drillon, Wiley-VCH: Verlag, 2002). The fitted *J* value was 12.56(3) cm^−1^ and *g* was 2.16(1), demonstrating the ferromagnetic interactions between W^V^ and Fe^II^_HS_.

Figure S1. Crystal structure of Fe^II^-SCO. Fe^II^-SCO is composed of one-dimensional coordination chains, wherein the FeII and WV ions are alternately arranged via cyanide bridges. These chains are linked by 1,4-bis(1H-imidazol-1-yl)benzene ligands, forming a three-dimensional metal-organic framework. (Notably, the three-dimensional framework structure does not necessarily imply the formation of three-dimensional magnetic order, as the ligands do not propagate magnetic order to adjacent chains. The magnetic order has been investigated that the Fe^II^_LS_-SCO crystal exhibits molecular nanomagnet behavior from short Fe(II)-W(V) fragment, whereas Fe^II^_HS_-SCO crystal exhibits single-chain magnet character, thus eliminating the possibility of three-dimensional long-range order.)


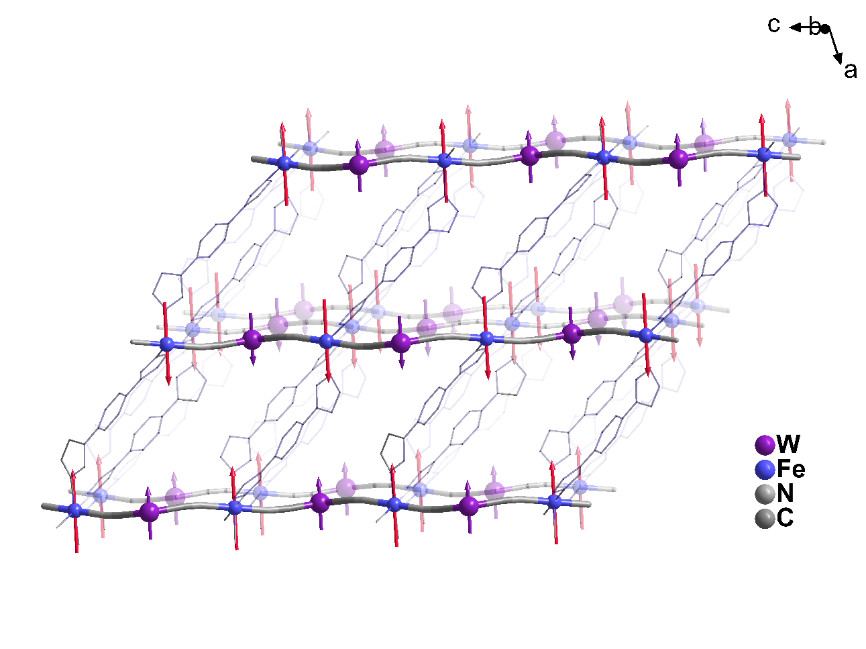


Figure S2. Optical image (a) and schematic illustration (b) of the device structure. The Fe^II^-SCO single crystal was set on two parallel antennas spaced 100 micrometers apart, and the molecular chain is perpendicular to the two antennas in the direction of spin-wave propagation. The two antennas were separately connected to the port 1 and the port 2 of the network analyzer and ground connected at the other ends so that the electric field component is nearly zero at the ends of the two antennas, and a maximum alternative magnetic field was excited near the crystal, which allowed us to maximize the magnons.

# Figure S3. The microwave spectrum S21 of the crystal-device with the 808 nm laser on and the 808 nm laser off at 10 K.

# Figure S4. The time-dependent Δ*_f_*_1_*_f_*_2_ with the 808 nm laser on and the 808 nm laser off at 10 K of the crystal-device.
